# X-linked hypophosphatemic rickets: a new mutation

**DOI:** 10.1590/2175-8239-JBN-2020-0027

**Published:** 2020-09-04

**Authors:** Patrícia Maio, Lia Mano, Sara Rocha, Rute Baeta Baptista, Telma Francisco, Helena Sousa, João Parente Freixo, Margarida Abranches

**Affiliations:** 1 Hospital do Espírito Santo de Évora, Évora, Portugal.; 2 Centro Hospitalar e Universitário de Lisboa Central, Hospital Dona Estefânia, Lisboa, Portugal.; 3 Hospital de Santarém, Santarém, Portugal.; 4 Centro Hospitalar e Universitário de Lisboa Central, Hospital Dona Estefânia, Unidade de Nefrologia Pediátrica, Lisboa, Portugal.; 5 Hospital de Vila Franca de Xira, Vila Franca de Xira, Lisboa, Portugal.; 6 Centro Hospitalar e Universitário de Lisboa Central, Hospital Dona Estefânia, Departamento de Genética Médica, Lisboa, Portugal.

**Keywords:** Rickets, Hypophosphatemic, Mutation, PHEX, Raquitismo hipofosfatêmico, Mutação, PHEX

## Abstract

Phosphopenic rickets may be caused by mutations in the *PHEX* gene (phosphate regulating endopeptidase homolog X-linked). Presently, more than 500 mutations in the *PHEX* gene have been found to cause hypophosphatemic rickets. The authors report a clinical case of a 4-year-old girl with unremarkable family history, who presented with failure to thrive and bowing of the legs. Laboratory tests showed hypophosphatemia, elevated alkaline phosphatase, normal calcium, mildly elevated PTH and normal levels of 25(OH)D and 1.25(OH)D. The radiological study showed bone deformities of the radius and femur. Clinical diagnosis of phosphopenic rickets was made and the genetic study detected a heterozygous likely pathogenic variant of the *PHEX* gene: c.767_768del (p.Thr256Serfs*7). This variant was not previously described in the literature or databases. Knowledge about new mutations can improve patient’s outcome. Genetic analysis can help to establish a genotype-phenotype correlation.

## Resumo

O raquitismo fosfopênico pode ser causado por mutações no gene PHEX (ligado ao X do homólogo da endopeptidase que regula o fosfato). Atualmente, mais de 500 mutações no gene PHEX causam raquitismo hipofosfatêmico. Os autores relatam um caso clínico de uma menina de 4 anos com histórico familiar sem relevância, que apresentou falha no crescimento e arqueamento das pernas. Os exames laboratoriais mostraram hipofosfatemia, fosfatase alcalina elevada, cálcio normal, PTH levemente elevado e níveis normais de 25(OH)D e 1,25(OH)D. O estudo radiológico mostrou deformidades ósseas no rádio e no fêmur. O diagnóstico clínico do raquitismo fosfopênico foi realizado e o estudo genético detectou uma provável variante patogênica heterozigótica do gene PHEX: c.767_768del (p.Thr256Serfs*7). Esta variante não foi descrita anteriormente na literatura ou nas bases de dados. O conhecimento sobre novas mutações pode melhorar o desfecho de pacientes. A análise genética pode ajudar a estabelecer uma correlação genótipo-fenótipo.

## Introduction

Phosphopenic rickets occurs as a result of inherited or acquired abnormalities in the proximal tubular handling of phosphorus^1^. X-linked hypophosphatemic rickets (XLH) is the most common inherited form of rickets with an incidence of 1:20,000 individuals^2^^-^ 5. This disorder is caused by inactivating pathogenic variants in *PHEX* gene (Phosphate regulating Endopeptidase homolog X-linked), which is located on chromosome locus Xp22.1 and contains 22 exons^6^. These inactivating variants result in excess circulating FGF-23 (fibroblast growth factor 23), that impairs renal phosphate reabsorption on proximal tubule cells via FGFR1 (fibroblast growth receptor 1) and its co-receptor KLOTHO.

Although XLH rickets is inherited in an X-linked dominant way, the severity of its manifestations is variable. The diagnosis of XLH is suspected based on clinical manifestations, laboratory abnormalities, and X-ray findings. It can be confirmed by the identification of a hemizygous (in males) or heterozygous (in females) pathogenic variant in *PHEX* by molecular genetic testing^7^^,^ 8. A positive family history can facilitate the diagnosis, but *de novo* mutations occur frequently^8^.

Clinical manifestations include growth retardation, abnormal bone mineralization, osteomalacia, bone pain, and deformity of the lower limbs (*genu varus* or *valgus*)^7^^,^ 9. Usually the birth length is normal, but the growth rate slows in infancy^7^. The clinical manifestations often become apparent in the first two years of life, especially when the child begins to walk, causing bowing of the legs and short stature.

Affected individuals may present dental malposition and periradicular abscesses due to defective dentin or enlarged pulp chambers and root canals^10^. Premature cranial synostosis can occur with dolichocephaly, parietal flattening, and frontal bossing^8^^,^ 11 ^,^ 12. Adults may present pseudofractures, osteoarthritis, osteophytes, or enthesopathy.

Laboratory findings include hypophosphatemia with hyperphosphaturia, normal serum calcium levels, normal or reduced calciuria, and normal 25 - (OH) - vitamin D levels. The serum levels of PTH are normal or mildly elevated, and plasmatic alkaline phosphatase is increased. There is resistance to high doses of vitamin D^13^. High serum levels of FGF-23 can be found^10^.

X-ray findings include deformities of the lower limb and widened, frayed, or cupped metaphyses^7^. In adult patients, calcifications of the tendons or ligaments can be present^7^.

In this report, we describe a patient with hypophosphatemic rickets as result of a novel likely pathogenic variant in *PHEX* gene.

## Case Description

**Figure 1:**
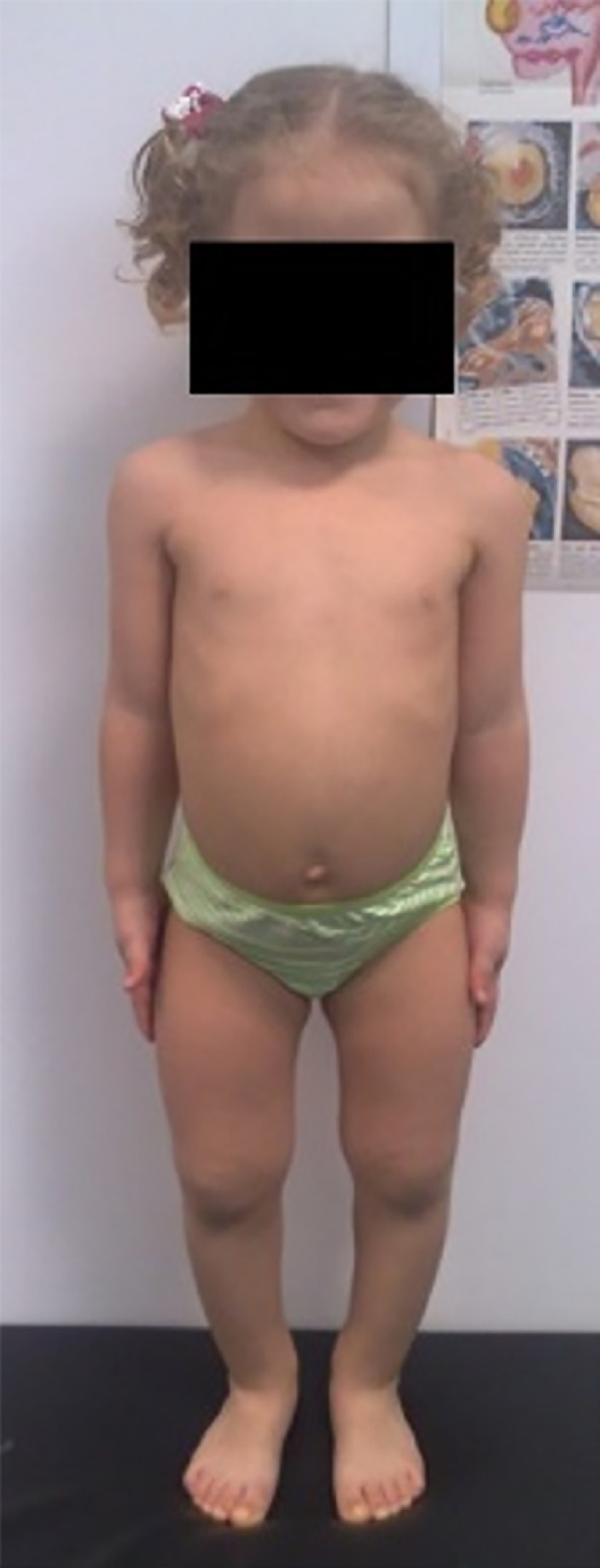
Proband at 4 years of age, presenting with bowed legs, bilateral *genu varum*, and enlarged wrists.

A 4-year-old female, with unremarkable family history, born at full term with adequate weight and length, presented with failure to thrive since the first year of life (height at the 5^th^ centile until 2 years old, and at the age of four below 5^th^ centile). Leg bowing was noted by the age of 18 months. On physical examination, she had frontal bossing, hyperlordosis, bowed legs (bilateral genu varum), thickened wrists, normal teeth and hair (Figure 1). No complaints of muscle pain were referred.

Blood workup showed hypophosphatemia (2.4 mg/dL), elevated alkaline phosphatase (495 U/L), normal calcemia, mildly elevated PTH (97.2 pg/mL; RR <68.3), and normal levels of 25-(OH)-vitamin D and 1.25-(OH)-vitamin D. The radiological evaluation showed bone deformities of the radius and femur.

As the diagnosis of phosphopenic rickets was made, she started treatment with calcitriol 125 mcg/day and phosphorus 2500 mg/day.

**Figure 2:**
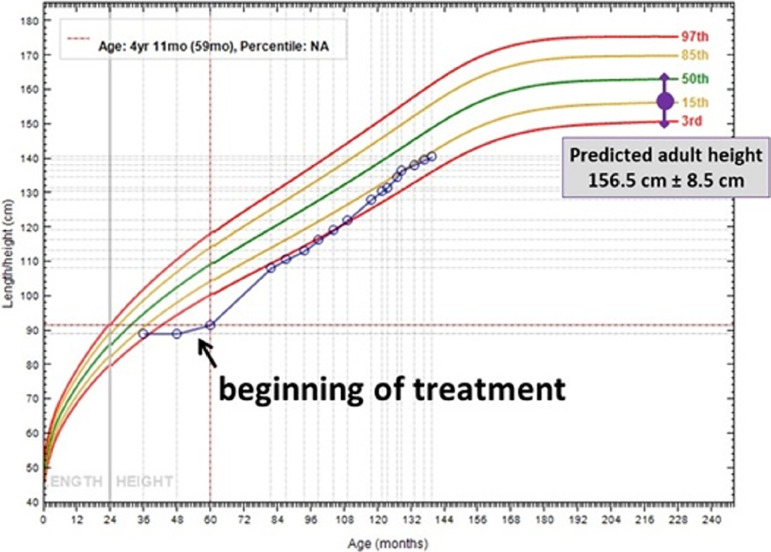
Evolution of the patient’s height.

Currently, at 11 years old, the patient has no clinical or radiographic signs of rickets. Bone age is in agreement with the chronological age and there was a considerable increase in growth rate (15th centile), which corresponds to her target height (Figure 2). Renal ultrasound shows incipient signs of nephrocalcinosis since the age of nine. Blood workup shows PTH 53.20 pg/mL, alkaline phosphatase 291 U/L, phosphatemia 2.7 mg/dL, and calcemia 10.2 mg/dL.

The genetic analysis detected a heterozygous likely pathogenic variant of the *PHEX* gene: variant c.767_768del (p.Thr256Serfs*7). This variant was not previously described in literature or databases. However, since it introduces a premature stop codon resulting in a truncated protein, which is a known mutational mechanism of XLH, this is very likely to be a pathogenic variant. The parent’s genetic study is still in progress.

## Discussion

The authors report the case of a girl with clinical, biochemical, and radiologic findings of phosphopenic rickets. The patient received treatment with phosphorus and calcitriol with growth improvement. The genetic study identified a novel likely pathogenic variant in *PHEX* gene, which produces a premature stop codon, resulting in a truncated protein.

A *PHEX* gene pathogenic variant was first described in 1995^14^. Presently, more than 500 pathogenic variants in this gene have been reported to cause XLH (HGMD professional 2019.4). Different gene defects including missence, nonsense, splice site, small and gross deletions, and insertions have been described in the literature, in the Human Gene Mutation Database, and in the PHEX mutation database^15^.

The PHEX gene encodes a membrane-bound endopeptidase that is expressed at the cell-surface membrane of osteoblast, osteocytes, odontoblasts, lung, liver, muscle and gonads^7^^,^ 10. *PHEX* pathogenic variants increase the production of FGF-23, which promotes a phosphaturic effect, leading to hypophosphatemia. Despite that, the pathophysiologic mechanism through which the *PHEX* loss of function variants increase the levels of FGF-23 is not fully understood^7^.

In patients with XLH, the renal phosphate wasting should be evaluated by calculating the tubular maximum reabsorption of phosphate per glomerular filtration rate (TmP/GFR)^13^.

Conventional treatment of children with hypophosphatemic rickets includes a combination of oral doses of phosphate preparations (four to five times a day) and active vitamin D analogs (calcitriol or alfacalcidol)^10^. Goals of treatment also include normalization of alkaline phosphatase and trying to maintain calciuria in the normal range, to avoid calcium deposition in the renal parenchyma; however, normalization of serum levels of phosphate is not a goal of conventional therapy, as it would be difficult to achieve and would also promote nephrocalcinosis^10^^,^ 13.

In this report, the patient present incipient signs of nephrocalcinosis since the age of nine. This adverse effect of treatment has been controlled with kidney ultrasonography and adjustments of therapy avoiding large doses of phosphate supplements and keeping normal calciuria levels. If necessary, the use of potassium citrate can help to prevent calcium precipitation, but it increases the risk of phosphate precipitation. Therefore, potassium citrate should be used with caution^13^. The therapy with burosumab should be considered if nephrocalcinosis worsens.

burosumab (Crysvita^®^), a fully human recombinant IgG1 monoclonal antibody directed at fibroblast growth factor 23 (FGF23) was approved by the European Medicines Agency (EMA) in February 2018 for the treatment of XLH with radiologic bone disease in children ≥1 year of age and in adolescents with a growing skeleton. It has also been approved by the US Food and Drug Administration (FDA) in April 2018 for the treatment of XLH in adults and children ≥1 year^13^^,^ 16. The starting dose is 0.4 mg/kg, with a maintenance dose of 0.8 mg/kg (up to a maximum dose of 90 mg) administered as a subcutaneous therapy once every 2 weeks^16^.

The decision to approve burosumab to treat XLH in adults and children were based in the results of several trials. Two open-label uncontrolled trials testing burosumab in 65 children aged 1-12 years with severe XLH demonstrated that in 12-16 months burosumab resulted in a statistically significant increase in TmP/GFR, with subsequent higher serum phosphate levels, higher 1,25(OH)2 vitamin D levels, a significant reduction in the severity of rickets, a remarkable improvement in physical ability, and a significant reduction in patient-reported pain and functional disability^13^.

In this case, burosumab was considered but postponed due to the successful response to conventional therapy.

In conclusion we report a novel, likely pathogenic, variant in *PHEX* gene in a girl with clinical, laboratory, and radiological findings of rickets. Genetic diagnosis is extremely important as it may determine precise treatment decisions and enable genetic counselling and genetic prenatal diagnosis^17^. Knowledge about the mutational spectrum of genetic diseases is important for better genotypic characterization and can improve the patient’s outcome. Genetic analysis can help to establish a genotype-phenotype correlation^13^.
